# Supplementary material for: KCa1.1 K+ Channel Inhibition Overcomes Resistance to Antiandrogens and Doxorubicin in a Human Prostate Cancer LNCaP Spheroid Model
Source: Int J Mol Sci. 2021 Dec 17;22(24):13553. doi: 10.3390/ijms222413553 (PMC8706449; doi:10.3390/ijms222413553)
Supplement: Supplementary file 1 [file ijms-22-13553-s001.zip › ijms-1508159-supplementary.pdf]

Supplementary Materials

Target name	Genbank accession number	Primer sequence		amplicon length (bp)
		Forward (5' to 3')	Reverse (5' to 3')	
$K_{Ca}1.1$	NM_001014797	GCCGGGTTCATCCATTTG	ACCAACGGTGGACATTGTGA	120
KCNMB1	NM_004137	AGCCCTTTGCCTGGGTGTAA	CAGGTGGCACTTGGATTCCT	121
KCNMB4	NM_014505	TCCCAGCCATTTACTTGCTATT	AGAACGCCCACCACAAATGT	131
LRRC26	NM_001013653	GCGCTGCGCCCGCTCTGCGC	GTCGGAAAAGGCAGTCAGG	120
LRRC38	NM_001010847	CAGACCTCTGCATCATCATTTTCT	CGTCTTCATCCTCCGCATCT	130
LRRC52	NM_001005214	ACAGGTGTGTGTGCTGTGCTCTA	CGAGCTCGTCTGGGTTTGAA	120
LRRC55	NM_001005210	GTGCTGGATTTGCACAACAACT	AACATGTCGGCTGGCACAT	120
FBXW7	NM_033632	TCACAAATGAGAGACAACATCATCA	ACAACGCACAGTGGAAGTATGC	120
MDM2	NM_002392	ATGAATCCCCCCCTTCCAT	TGAGTTTTCCAGTTTGGCTTTCT	120
CRBN	NM_016302	GCCTATCGAGAGAACAGGATTTT	CACTTTAGCTTGCTGGATTCCA	120
MDR1	NM_001348945	TCACCATGGATGAGATTGAGAAA	TGGCGATCCTCTGCTTCTG	128
MDR3	NM_000443	CCACAGATGCTGCCCAAGT	GGGTTAACTGCCAACCGTAGAT	120
MRP1	NM_004996	AGGCGAGTGTCTCCCTCAAA	TCCTCACGGTGATGCTGTTC	120
MRP2	NM_000392	CTCAAGAACCTGCTGGCCTTT	ATTGTTGCTATCCACCAGGACAT	120
MRP3	NM_003786	GGCTGGGCTGATGTTCCTG	GCCTTCCTGTAGATGACACCCATG	123
MRP4	NM_005845	CTGTGCGGCTGACGGTTAC	GGTTGCGCTGTGATATCTCATC	120
MRP5	NM_005688	CCCAGGCAACAGAGTCTAACCT	CACCGGTTCGGTAATTCAAT	120
MRP6	NM_001171	TTAGACGCGAGAGGTCCATCA	CGTATTGGATGCTGTCCTTTCC	129
ABCG1	NM_016818	AGGGACTCGGTCCTGACACA	GAAGCCGGAGTTGCTCAAGA	120
ABCG2	NM_004827	CGATATGGATTTACGGCTTTGCA	CCAAATATTCTTCGCCAGTACATG	121
NANOG	NM_024865	CCTTCCTCCATGGATCTGCTTA	CTTGACCGGGACCTTGTGTTC	120
KLF4	NM_001314052	ACGGCTGTGGATGGAAATTC	GGTGGTCCGACCTGGAAAAT	120
CD44	NM_000610	TACAAGCACAATCCAGGCAACT	TGGGAGTCTTCTTTGGGTGTTT	120
AR	M20132	GCCTTGCTCTCTAGCCTCAA	AGGAGTACTGAATGACAGCC	127
Nrf2	NM_006164	TGCCCCTGGAAGTGTCAAAC	TCACATTGGGCATCATGCA	120
IL-1β	NM_000576	AACGAGGCTTATGTGCACGAT	TCCCTGGAGGTGGAGAGCTT	120
SKP2	NM_005983	AGTGAGAACATCCCCCAGGAA	TAGCTTAGGCCTGCGGACAA	120
RNF6	NM_005977	GAGCTAACGCTTCACGCACTAA	TTCTAGCCCCATTTTCCCAAA	120
SIAH2	NM_005067	GGCTGTGGACTGGGTGATG	GTGCCAATGAGCAGGACGAT	118
USP14	NM_005151	ATTGCAACAGAAATTGGAAGCA	TCAACACCGAAGAACTGATCGA	120
UBE3A	NM_130838	TGCTGCTTCGAAGTGCTTGA	AGTGTCAGCTCGCTGGACTCA	120
NRIP	NM_018442	CTGTTGCTATTTGCCCACCAA	CTCGACCTGCATAATTCCCTGTA	120
ACTB	NM_001101	AGGCCAACCGCGAGAAGATG	GCCAGAGGCGTACAGGGATA	101

Scheme S1. PCR primer information for real-time PCR.

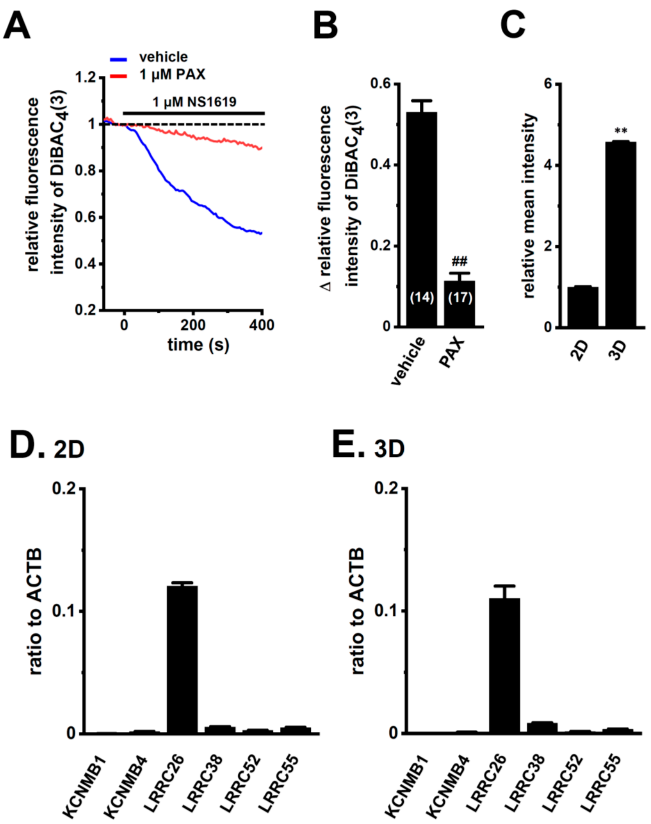

Figure S1. Inhibition of NS1619-induced hyperpolarizing responses by a $K_{Ca}1.1$ inhibitor increased the expression level of the $K_{Ca}1.1$ protein at the plasma membrane in 3D-cultured LNCaP cells and the expression of $K_{Ca}1.1$ auxiliary β and γ subunits in 2D- and 3D-cultured LNCaP cells. (A): Time course of the voltage-sensitive fluorescent dye imaging of $K_{Ca}1.1$ activator (1 μM NS1619)-induced hyperpolarizing responses in isolated cells from LNCaP spheroids. Cells were pre-incubated with vehicle (blue) or PAX (1 μM) (red) for 5 min. The fluorescent intensity of $DiBAC_4(3)$ before the application of NS1619 is expressed as 1.0. Images were measured every 5 s. (B): Summarized results of NS1619-induced hyperpolarizing responses. Cell numbers used in experiments are shown in parentheses. The values for fluorescent intensity were obtained by measuring the average for 1 min (12 images). (C): Fixed, non-permeabilized LNCaP cells were stained with an Alexa Fluor 488-conjugated anti-$K_{Ca}1.1$ (extracellular) antibody, and mean fluorescence intensities were measured using flow cytometry. The intensities in '2D' were expressed as 1.0 (n = 4 for each). (D,E): Real-time PCR examination of $K_{Ca}1.1$ auxiliary β and γ subunits (KCNMB1, KCNMB4, LRRC26, LRRC38, LRRC52, and LRRC55) in 2D- (D) and 3D-cultured (E) LNCaP cells (n = 4 for each). Expression levels were shown as a ratio to ACTB. **: $p < 0.01$ vs. '2D' and $^{##}$: $p < 0.01$ vs. vehicle control.

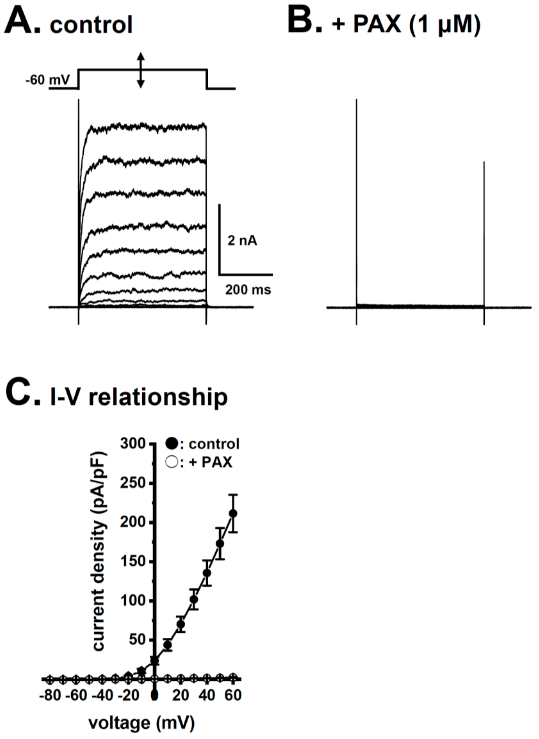

Figure S2. Effects of the K_{Ca}1.1 inhibitor, PAX, on depolarization-induced outward K^+ currents in 3D-cultured LNCaP cells. (A,B): Currents were elicited by a 500 ms depolarizing voltage step between −80 and +60 mV from a holding potential (−60 mV) with 10-mV increments in 3D-cultured LNCaP cells (A, control). The application of 1 μM PAX markedly reduced outward currents (B). (C): Current density-voltage relationship for outward K^+ current amplitude in control (●) and PAX-treated (O) groups (n = 8).

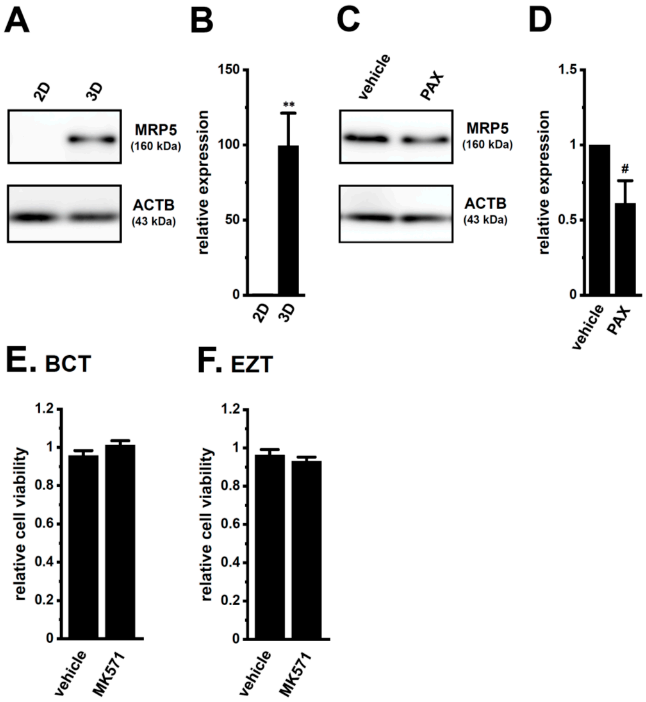

Figure S3. Increase in MRP5 protein expression by LNCaP spheroid formation, and its reversal by the PAX treatment in LNCaP spheroids. (A,C): MRP5 protein expression in protein lysates from the lipid-raft-enriched fractions of 2D- and 3D-cultured LNCaP cells (A) and vehicle- and PAX-treated, 3D-cultured LNCaP cells (n = 4 for each) (C). Blots were probed with anti-MRP5 (approximately 160 kDa, upper panel) and anti-ACTB (43 kDa, lower panel) antibodies. (B,D): Summarized results were obtained as the optical densities of MRP5 and ACTB band signals, respectively (n = 4 for each). After compensation for the optical density of the MRP5 protein band signal with that of the ACTB signal, the MRP5 signal in '2D' (B) or 'vehicle' (D) was expressed as 1.0 (n = 4 for each). (E,F): Effects of the treatment with 10 μM BCT and EZT for 48 h on the viability of vehicle- and 10 μM MK571-cotreated LNCaP spheroids (n = 5 for each). **: $p < 0.01$ vs. '2D' (B); $^{\#}$: $p < 0.05$ vs. vehicle control (D).

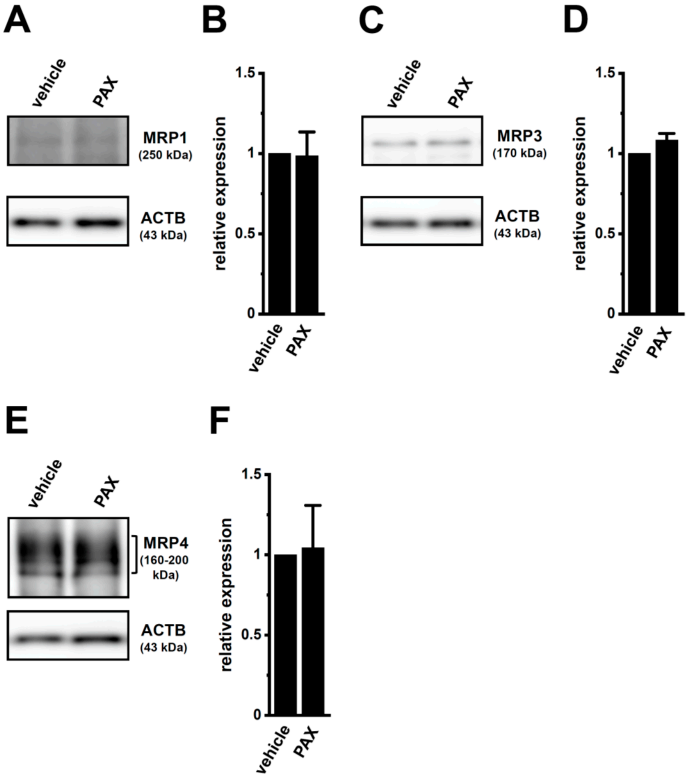

Figure S4. No significant changes in MRP1, MRP3, and MRP4 protein expression by the PAX treatment in LNCaP spheroids. (A,C,E): MRP1, MRP3, and MRP4 protein expression in protein lysates from the lipid-raft-enriched fractions of vehicle- and PAX-treated, 3D-cultured LNCaP cells (n = 4 for each). Blots were probed with anti-MRP1 (approximately 250 kDa, upper panel) (A), anti-MRP3 (approximately 170 kDa, upper panel) (C), anti-MRP4 (160–200 kDa, upper panel) (E), and anti-ACTB (43 kDa, lower panel) (A,C,E) antibodies. (B,D,F): Summarized results were obtained as the optical densities of MRP1, MRP3, MARP4, and ACTB band signals, respectively (n = 4 for each). After compensation for the optical density of the MRP protein band signal with that of the ACTB signal, the MRP signal in 'vehicle' was expressed as 1.0 (n = 4 for each).

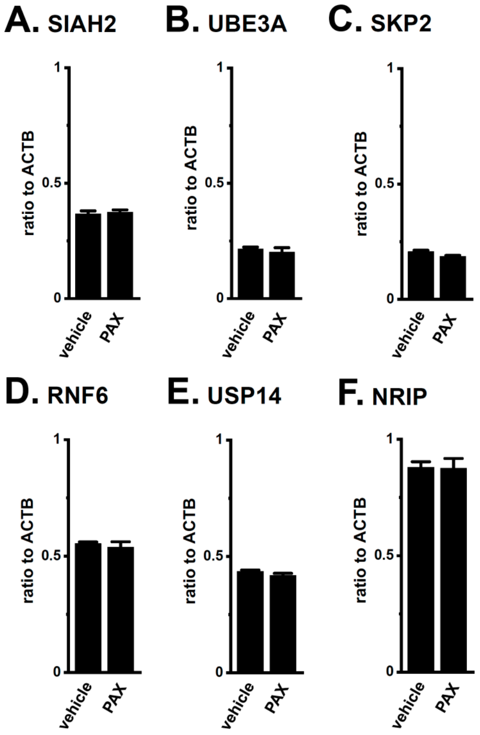

Figure S5. Effects of the treatment with PAX for 24 h on expression levels of ubiquitin E3 ligases, SIAH2, UBE3A, SKP2, RNF6, USP14, and NRIP in LNCaP spheroids. A-F: Real-time PCR examination of SIAH2 (A), UBE3A (B), SKP2 (C), RNF6

(D), USP14 (E), and NRIP (F) in vehicle- and 10 μM PAX-treated, 3D-cultured LNCaP cells (for 24 h) (n = 4 for each). Expression levels were shown as a ratio to ACTB.

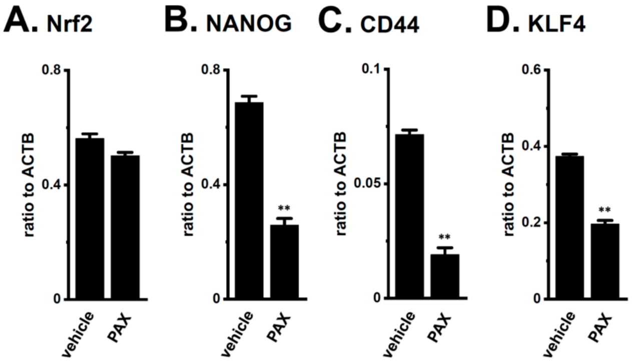

Figure S6. Effects of the treatment with PAX on expression levels of Nrf2, NANOG, CD44, and KLF4 in LNCaP spheroids. (A–D): Real-time PCR examination of Nrf2 (A), NANOG (B), CD44 (C), and KLF4 (D) in vehicle- and 10 μM PAX-treated 3D-cultured LNCaP cells (for 24 h) (n = 4 for each). Expression levels were shown as a ratio to ACTB. **: $p <$ 0.01 vs. vehicle control.
